# Prof. Ludger Wessjohann: A Lifelong Career Dedicated to a Remarkable Service in “Natural Products Sciences”

**DOI:** 10.3390/ijms23105440

**Published:** 2022-05-13

**Authors:** Hidayat Hussain

**Affiliations:** Department of Bioorganic Chemistry, Leibniz Institute of Plant Biochemistry, Weinberg 3, 06120 Halle, Germany; hidayat.hussain@ipb-halle.de

It is a great honor and a pleasure for me to serve as Guest Editor for this Issue of the “*International Journal of Molecular Sciences*”, dedicated to our mentor and colleague, Professor Dr. Ludger Wessjohann on the occasion of his 60th birthday. This Special Collection honoring Professor Wessjohann represents an excellent opportunity to celebrate not only a remarkable chemist, but also a great man.

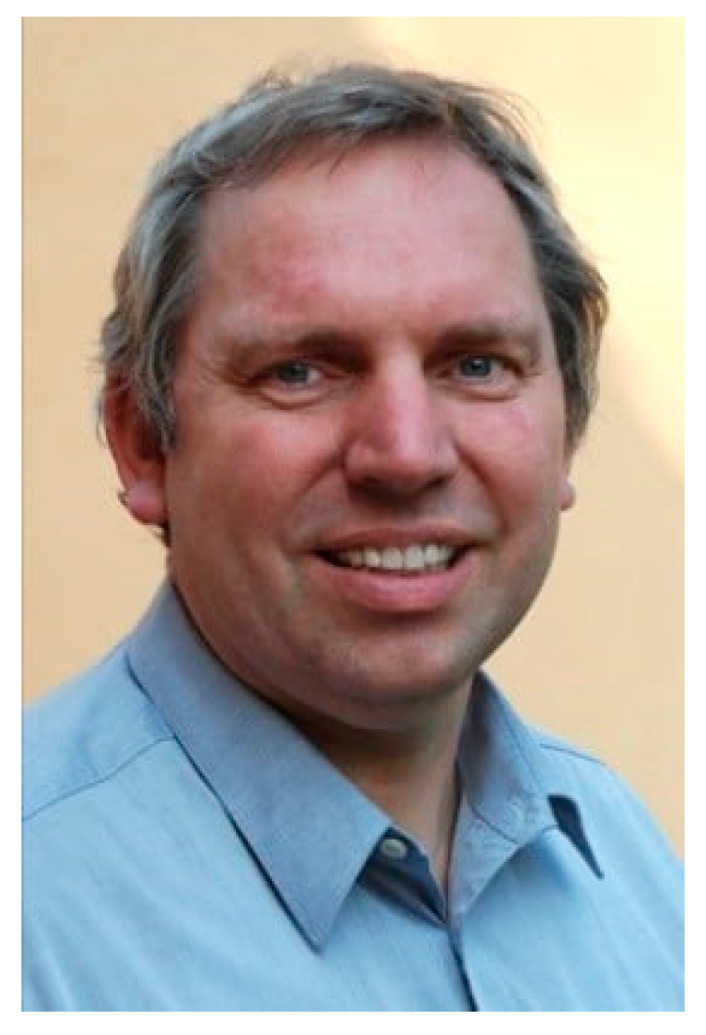



Professor Wessjohann studied chemistry in Hamburg (Germany), Southampton (UK), and Oslo (Norway, Prof. Skattebøl). He earned his doctorate in 1990 with Prof. Armin de Meijere in Hamburg. After a short period as a lecturer in Brazil, he became a postdoctoral Feodor Lynen fellow of the Alexander von Humboldt Foundation with Prof. Paul Wender at Stanford University (USA), working on the total synthesis of Taxol^®^. After an assistant professorship in Munich (LMU, 1992–1998), he was appointed to the Chair of Bioorganic Chemistry at the Vrije Universiteit Amsterdam (NL), working on organometallic chemistry and biocatalysis. Since 2000, he has been the director of the Department of Bioorganic Chemistry at the Leibniz Institute of Plant Biochemistry (IPB) in Halle (Germany) and concurrently holds the chair of Natural Product Chemistry at the Martin Luther University of Halle-Wittenberg. From 2010–2017 he served as the Managing Director of the IPB (www.ipb-halle.de (accessed on 19 April 2022)).

Among others, he has been honored with the following awards: Leibniz Biotechnology Process of the Year (2019); Leibniz Bioactive Compound of the Year (2016 and 2018); Hugo Junkers Innovation Prize, State of Saxony-Anhalt, Germany (2014 and 2018); visiting Professor (invited) of King Saud University, Saudi Arabia; IQ Innovation Prize, City of Halle (2008); Microsoft IT-Founders Prize (Ontochem); winner of the business plan competition Sachsen-Anhalt (2006); and Feodor Lynen Fellowship (Alexander von Humboldt Foundation, 1990–1991). He is also a foreign member of the Brazilian Academy of Sciences, Brazil, and an honorary member of the Argentinean Society of Synthetic Organic Chemistry, Argentina, and in 2019 was one of the thirteen foreign scientific advisors to the Colombian government’s “Misíon des Sabios”, with the task of drafting a future scientific agenda for the whole country. 

He is a speaker at the Leibniz Science Campus Halle on “Plant-Based Bioeconomy” and the cofounder of the yearly “International Bioeconomy Conference” held at the National Academy of Sciences Leopoldina, in Halle. Additionally, he has been the organizer and co-organizer of several national and international conferences and conference series, e.g., the International Conference on the Chemistry of Selenium and Tellurium, the Multicomponent Reactions Conference, and the Brazilian Meeting of Organic Chemistry, to name but a few. Moreover, he is a member of steering committees and advisory boards of companies and organizations such as the national scientific advisory board of the Colombian government in the fields of bioeconomy, biotechnology, and environment; a board member of the European Federation of Biotechnology, Plant, Agriculture & Food Division; and the Dechema Fachgruppe Biotransformationen. Furthermore, Professor Wessjohann has served on numerous journal editorial boards, selection committees of foundations and science organizations (e.g., Alexander von Humboldt Foundation, DAAD, Finnish Academy of Sciences, Dutch Science Foundation). 

He has authored or coauthored over 560 research articles (up to the end of 2021), including >20 reviews and >30 patent families issued or published. In addition, he has over 15000 citations, including a GS H-index of 63. Notably, according to the Stanford University database, his name is included in the top two percent of the most-cited scientists in various disciplines for 2020 and 2021. Moreover, he is the cofounder of six companies. He has been the major and/or thesis advisor for over 50 graduate students and has also directly supervised some 100 postdoctoral and visiting scholars. Furthermore, he has presented numerous research seminars at international and national scientific meetings in more than 30 countries. He has established very strong collaboration with scientists at several universities and academies in Cuba, Brazil, Colombia, Mexico, Vietnam, and some Arabic countries. 

Professor Wessjohann’s main research areas include molecular interactions in bioorganic and medicinal chemistry: (i) natural products—from isolation to total synthesis and bioactivity (incl. metabolomics, proteomics); (ii) biocatalysis and enzymatic reactions; (iii) new synthetic methods, combinatorial and medicinal chemistry (multicomponent reactions (MCR), pept(o)ides, macrocycles); and (iv) chemoinformatics. The main sources of his natural product discovery work are plants and higher fungi (“mushrooms”). His application covers the fields of anticancer agents, neuroactives, anti-infectives, and aroma compounds (flavor and fragrance), as well as plant protectants and phytoeffectors. He once said: “Coming originally from synthetic anticancer agents, natural product discovery quickly forced me to look more broadly. When you research organismic constituents, you do not know what they will be good for initially. Additionally, working in an institute with so many plant competent biologists, looking for compounds to increase plant productivity is a natural development—and it is a field underdeveloped in academic chemistry.”

Professor Wessjohann’s periods of service at IPB (since 2001) have been times of outstanding productivity and very dedicated service to the natural product research, academia, and health communities. Based on his remarkable research output, he is recognized as an internationally outstanding investigator and inspirational leader of collaborative research projects.

Prof. Dr. Wessjohann’s research work, throughout his long career, has covered both plant-, fungi-, and coral-derived natural products developed toward drug candidates and agrochemical and food products, as can be seen from his extensive publication record. In addition, he has pioneered a number of synthetic methods for the synthesis of peptides, peptoids, and other natural products. However, beyond the analysis and discovery of phytochemicals, Ludger has pioneered many spectacular advances in the synthesis of fascinating natural products, intermediates, and derivatives. These scientific efforts led to the discovery of the second generation of tubulysins, the “tubugi” derivatives. Notably, tubugi-1 illustrated cytotoxic activity similar to that of tubulysin A and its activity was 30 fold higher than paclitaxel, with better stability and synthesizability than the parent compound [1]. Tubulysins are tetrapeptides, which constitute an intriguing natural product family with potent antimitotic properties [2]. Wessjohann’s derivatives are especially suitable for targeting approaches as drug conjugates, e.g., with peptides and antibodies. Most excitingly, they enhance innate immunity against cancer and exhibit an atypical apoptosis mechanism. The synthetic tubulysin derivative, tubugi-1, improves the innate immune response by macrophage polarization, in addition to its direct cytotoxic effects in a murine melanoma model [3]. 

Total synthesis of natural products plays a remarkable role in achieving unambiguous structural confirmation, absolute configuration determination, and access to derivatives. Notably, his innovative syntheses enabled his teams to get sufficient natural product material for preliminary and detailed biological and pharmacological investigations, along with the synthesis of derivatives for (Q)SAR studies and improved application profiles. To pick out just a few, the Wessjohann group accomplished the total syntheses of hygrophorones, cordyheptapeptide A [4], or, in 2013, epothilone D [5]. Epothilones are anticancer macrolides, which possess various remarkable advantages in comparison to Taxol (paclitaxel) and Taxotere, which are among frontline anticancer agents. The Wessjohann group also reported the total first ever total synthesis of tubulysins (U and V) [6] and of tubulysin B [7]. Some other interesting molecules are (-)-julocrotine, viridic acid (tetrapeptide), selancins A and B (acylphloroglucinols), and empetrifranzinans A and C (acylphloroglucinols). 

Notably, the Wessjohann lab is working on the applications of multicomponent reactions (MCRs) in the synthesis of natural and pseudo-natural products (in particular peptides and peptoids), which have been the subject of numerous publications throughout his career. It is noteworthy that the Wessjohann group is the world leader of synthesizing the peptoid backbones and macrocycles via Ugi and Passerini MCR reactions [8]. In addition, the group established the (cyclo-)-ligation and stapling of peptides with concomittant functionalization of the side chain [9] and backbone [10] via solution phase or on-resin MCR reactions. It is of note that the Wessjohann group established a MCR approach for the installation of turn-inducing moieties that facilitate the macrocyclization [11] along with ligation of peptides [12].

Prof. Wessjohann established an independent research program at IPB, involving the search for bioactive natural products, with well over a hundred new compounds described or patented, e.g., along with neuroscientists, he exploited the potential of *Rhodiola rosea* against Alzheimer disease and isolated ferulic acid eicosyl ester as a memory enhancer. Notably, this natural product used as nutraceutical (dried root material from *Rhodiola rosea*) possesses significant learning and memory enhancement properties in larval Drosophila and also partially compensates for age-related memory decline in adult flies [13], mice, and men. Another exemplary topic concerns natural sweetening and taste modifying agents from plants. His group isolated two sweet-tasting dammarane-type glycosides, balansins A and B, from *Mycetia balansae* Drake (Rubiaceae). Both balansins A and B demonstrated sweetening properties and their sweetness potencies at 0.1% and 0.2%, respectively, were equal to that of sucrose [14]. 

At the IPB, the Wessjohann group developed a process which they named “reverse metabolomics”, which was employed to ultimately link many biological effects (e.g., taste sensory data) with metabolic profiles. The process is based on the correlation of chromatographic and spectroscopic profiles of partial or total extracts, metabolite profiles in particular, with biological activity profiles by chemo-informatic protocols, using activity correlation analysis (AcorA) [15]. In addition, this esteemed research group developed the first comparative metabolomics approach for the assessment of secondary metabolites of common medicinal plants across variety and species borders in the context of their genetic diversity, phylogeny and growth habitat, or processing, so as to set a framework for its authentication and quality control analysis, at a time when metabolomics is otherwise concentrated on single species only [16]. His work on the multiplex metabolic profiles of licorice and *Glycyrrhiza* species became one of the most cited papers in medicinal/taste plant metabolomics [17]. He also was the first to use 2D-NMR in metabolic profiling (of hops) [18].

Recently, he applied smart data analyses and machine-learning to bio- and chemoinformatics to analyze the complete published knowledge of the plant natural products chemistry of a whole country (Indonesia; specifically, Java), with thousands of plants, habitats, and applications, based on over 750,000 papers and database entries for the region, thereby correlating phylogeny with bioactivities, pinpointing “hot” and “cold” clades useful for a certain purpose, e.g., antibiotic potential, and the “white spots” of current research [19]. Wessjohann was a pioneer in bringing difficult enzymatic reactions into organic synthesis, e.g., regioselective aromatic prenylation [20] or recently hydroxylation [21]. He established various biocatalysis protocols, including the ligation of coenzyme A-conjugates with cinnamic acids, and this methodology has found an application in biocatalytic cascades, e.g., to vanillin and its analogs, which is the world’s most-used flavoring agent [22].

Indeed, Professor Wessjohann has always been a highly inventive, creative, and stimulating mentor in encouraging his young students, along with collaborators, to expand their knowledge and vision in cross-border science fields via seminars, conferences, and critical scientific discussions in group seminars. Of note is his great intuition and ability to discuss everything about science with competence, creativity, and intelligence, which has undoubtedly allowed him to build and establish a remarkable work environment, in which a large and highly international and interdisciplinary group of coworkers has arisen, who, after leaving the group, continue to work in reputed institutions and universities (Figure 1). Of comparable importance is his establishment of a large school with many alumni, some of his Ph.D. students, posdoctoral fellows, and guest scientists who are shown in his academic family tree in Figure 1. Many of his former students are now professors around the world, with more than a dozen in Brazil alone. Indeed, his passion for honest science, work, and intriguing scientific ideas have inspired entire generations of scientists around the globe, as illustrated by the contributions to this outstanding Special Collection.

We are most grateful to Glinda He and the editorial team of the “IJMS” for their help in preparing this Special Collection and to Ines Stein, Prof. Wessjohann’s assistant, for their valuable help. Notably, we thank all the chemists who contributed to the collection, demonstrating their esteem and affection for Professor Wessjohann. On this special occasion, I would like to join Ludger’s colleagues, students, coworkers, collaborators, and friends in congratulating him on this 60th birthday. We all wish for him that he both continues and enjoys his scientific endeavors in good health for the coming years.

## Figures and Tables

**Figure 1:**
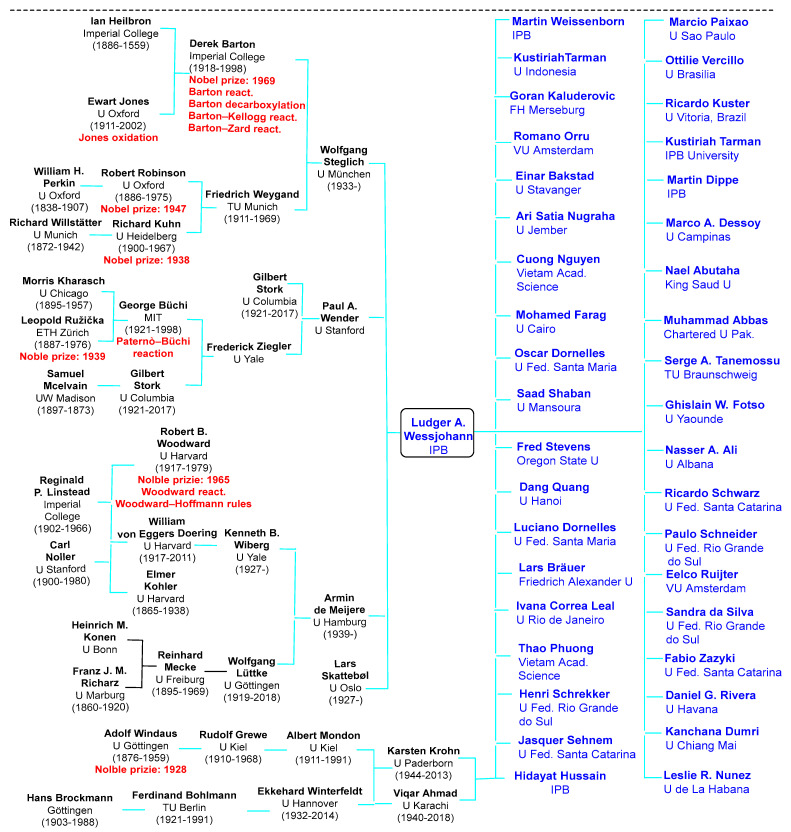
Ludger Wessjohann’s academic family tree; only some of the Ph.D. students and postdoctoral fellows he has hosted and supervised and who eventually became university professors are considered; all researchers associated with him would exceed the limits of this compilation.
